# Age-Related Different Relationships between Ectopic Adipose Tissues and Measures of Central Obesity in Sedentary Subjects

**DOI:** 10.1371/journal.pone.0103381

**Published:** 2014-07-22

**Authors:** Valeria Guglielmi, Luciano Maresca, Monica D'Adamo, Mauro Di Roma, Chiara Lanzillo, Massimo Federici, Davide Lauro, Paolo Preziosi, Alfonso Bellia, Paolo Sbraccia

**Affiliations:** 1 Department of Systems Medicine, University of Rome “Tor Vergata”, Rome, Italy; 2 Diagnostic Imaging Department, Policlinico Casilino, Rome, Italy; 3 Cardiology Department, Policlinico Casilino, Rome, Italy; University of Leicester, United Kingdom

## Abstract

Accumulation of fat at ectopic sites has been gaining attention as pivotal contributor of insulin resistance, metabolic syndrome and related cardiovascular complications. Intermuscular adipose tissue (IMAT), located between skeletal muscle bundles and beneath muscle fascia, has been linked to physical inactivity, ageing and body mass index, but little is known about its relationship with the other AT compartments, in particular with increasing age. To address this issue, erector spinae IMAT, epicardial (EAT), intraabdominal (IAAT) and abdominal subcutaneous adipose tissue (SAT) were simultaneously measured by Magnetic Resonance Imaging (MRI) and related to waist circumference measurements and age in 32 sedentary subjects without cardiovascular disease (18 men; 14 women; mean age 48.5±14 years). Fasting glucose, triglycerides and HDL-cholesterol were also assessed. We observed that, after dividing individuals according to age (≤ or >50 years), IMAT and EAT depots were significantly more expanded in older subjects (63.2±8.3 years) than in the younger ones (38.4±5.2 years) (p<0.001). Overall, both IMAT and EAT showed stronger positive associations with increasing age (β = 0.63 and 0.67, respectively, p<0.001 for both) than with waist circumference (β = 0.55 and 0.49, respectively, p<0.01 for both) after adjusting for gender. In addition, the gender-adjusted associations of IMAT and EAT with waist circumference and IAAT were significant in individuals ≤50 years only (p<0.05 for all) and not in the older ones. In contrast, no age-related differences were seen in the relationships of IAAT and SAT with waist circumference. Finally, serum triglycerides levels turned out not to be independently related with ectopic IMAT and EAT. In conclusion, the expansion of IMAT and EAT in sedentary subjects is more strongly related to age than waist circumference, and a positive association of these ectopic depots with waist circumference and IAAT amount can be postulated in younger individuals only.

## Introduction

The regional distribution of adipose tissue (AT) plays an important role in the development of metabolic and cardiovascular diseases [1]. Indeed, recent studies have suggested that the location [2] and inherent properties [3], [4] of excess AT, rather than total body adiposity, influence the autocrine, paracrine and endocrine effects of AT.

Whereas AT stored in subcutaneous depots is able to buffer the energy excess and to protect against the development of the metabolic syndrome (MS), intraabdominal adipose tissue (IAAT) is part of a more complex phenotype including a dysfunctional subcutaneous adipose tissue (SAT) and triacylglycerol deposition at ectopic, undesirable sites such as liver [5], heart and skeletal muscle [6]. Excess IAAT accumulation and AT stored in ectopic locations are closely related to clustering cardiometabolic risk factors like chronic inflammation, liver insulin resistance, hypertriglyceridemia and increased free fatty acid availability, presence of small, dense LDL particles, and reduced HDL cholesterol levels [3], [7].

Thus, ectopic adipose depots resulting from this defect in energy partitioning, have been gaining attention as potential and “regional” contributors to insulin resistance and obesity comorbidities.

At present, epicardial adipose tissue (EAT), namely the ectopic fat located subepicardically around both ventricles and along the coronary arteries, is reported to correlate with BMI, waist circumference and IAAT [8], [9], and to contribute independently to the development of coronary artery disease [10].

So far, few studies have focused on intermuscular adipose tissue (IMAT), which includes the visible storage of lipids in adipocytes located between the muscle fibers (also termed intramuscular fat) and between muscle bundles (literally intermuscular) [11], [12], [13].

IMAT is unambiguously linked with increase in body weight [14], physical inactivity or muscle disuse [15], and ageing [14], [16]. Although recent studies suggest that IMAT accumulation may be primarily the result of inactivity or muscle impairments rather than ageing *per se* [17], [18], therefore reflecting the importance of physical activity intervention even in the elderly, the potential relationship between central adiposity and IMAT accumulation, especially across different ages, is not fully understood.

Moreover, although IMAT accumulation has been shown to be significantly associated with insulin resistance [19], [20] and increased risk of type 2 diabetes [21], it is still unclear whether or not IMAT can play an intermediary role in developing insulin resistance, beyond its mere significance as marker of metabolic abnormalities.

Comparative studies demonstrated that magnetic resonance imaging (MRI), which is a well-established, validated method for estimation of AT and identification of its compartments [22], [23], has a higher sensitivity for identifying IMAT than computed tomography, because, not being density based, provides estimates not influenced by low density intra-myocellular lipids-enriched lean tissue [13].

According to this background, the primary aim of our study was to investigate the age-related association of erector spinae IMAT with adiposity measures and gender, in a sample of sedentary subjects. We additionally analyzed the relationships of MRI-assessed IMAT with EAT, IAAT, abdominal SAT and with several common cardiometabolic risk parameters.

## Materials and Methods

### Study population

We enrolled 32 patients (18 men; 14 women) undergoing cardiovascular MRI for arrhythmias (frequent ectopic ventricular and sopraventricular beats) in order to exclude genetic cardiomyopathy (hypertrophic cardiomyopathy, arrhythmogenic cardiomyopathy and dilated cardiomyopathy) or acquired cardiac disease (myocarditis, ischemic cardiac disease or inflammatory and autoimmune diseases). All subjects, before undergoing MRI, had been preliminarly evaluated by 12 leads electrocardiogram (EKG), 2D echocardiography, Holter EKG and stress test. Coronary artery disease was excluded by maximal negative stress test, or whenever indicated by coronary computed tomography angiography or coronary angiography. Inclusion criteria were: no pathological findings at the preliminary cardiologic evaluation and no pathological evidence revealed by cardiac MRI assessment; clinical history of normal fasting glucose; being non-smokers; having stable weight and dietary habits for ≥6 months prior to the study; having sedentary habits (based on self-report of no participation in vigorous routine or structured exercise). We excluded subjects affected by neoplasms, liver disease, renal insufficiency or any other severe systemic disease.

Waist circumference was recorded as the average of two measurements while the patients were standing, at midpoint between the lowest rib and the iliac crest. Body mass index (BMI) was calculated by dividing the weight (in Kg) by the square of height (in meter). Blood pressure (BP) was measured in the sitting position, with a standard, appropriately sized sphygmomanometer cuff. Three measurements were averaged to calculate systolic and diastolic BP.

Blood samples were obtained after overnight fast and glucose, HDL-cholesterol, triglycerides were assessed by routine laboratory techniques. MS was diagnosed according to IDF criteria [24].

Written informed consent was obtained from each patient included in the study. The study was approved by the Ethical Committee of the Fondazione Policlinico Tor Vergata (Rome), and it conforms to the principles of the Declaration of Helsinki.

### Magnetic Resonance Imaging

All patients underwent a standardized protocol including quantification of EAT volume during cardiac MRI and measurements of IMAT of rectus spinae, IAAT and SAT areas by two single slice detections at L3–L4 and L4–L5 level. MRI data were obtained with a Philips Intera 1.5 Tesla Achieva (Eindhoven, The Netherlands) scanner. Image analysis of EAT, IAAT and SAT was performed off-line using a stand-alone work station (Extended MR WorkSpace 2.6.3.4, 2012 Philips Medical System). SliceOmatic software (version 4.2; TomoVision, Montreal, Quebec, Canada) was used to analyze images of IMAT. To assess inter-observer reproducibility, a second independent observer repeated measurements in each dataset using the same conventions.

#### EAT volume

**Figure 1:**
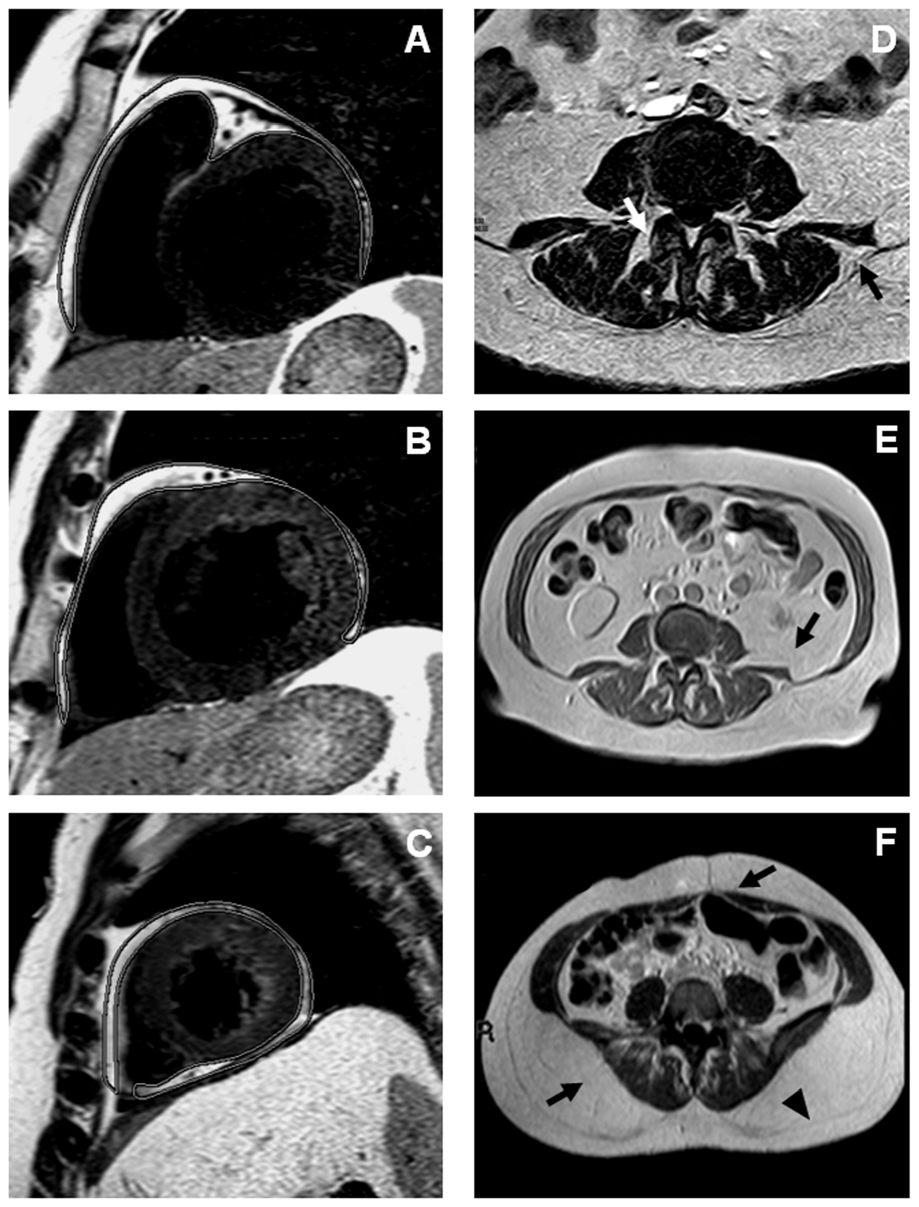
MRI measurements of adipose tissue depots. (A) Volumetric assessment of EAT. The contours of EAT were outlined in end-diastolic images of short-axis views covering the whole left and right ventricle. (B) IMAT of the erector spinae muscles at L3–L4 disk level. IMAT is visible between muscle groups (white arrow) and beneath the muscle fascia (black arrow). Abdominal L3–L4 (C) and L4–L5 (D) MRI scans with representation of IAAT and SAT respectively (black arrows). The black arrow head indicates the *fascia trasversalis* dividing SAT into a deep and a superficial layer.

For the assessment of EAT, we used a black blood prepared T2-weighted multislice to obtain a transversal 4-chamber view and short-axis images. Images parameters were as follows: time of repetition [25]  = 1600 ms, time to echo (TE) = 70 ms, slice thickness  = 4 mm, interslice gap (GAP)  = 2 mm and field of view (FOV) = 450 mm. EAT only included fat between the myocardial border and the internal visceral layer of the pericardium. Areas of EAT were traced manually on consecutive end-systolic short-axis images beginning at the mitral valve and ending at the last slice containing cardiac tissue. The areas obtained for each slice were added together and multiplied by slice thickness to yield EAT volume (Fig. 1 A–C) [8].

#### IMAT area

A transverse section was obtained at L3–L4 disk level to measure IMAT of the erector spinae muscles (including the multifidus, longissimus, and iliocostalis). The erector spinae musculature was chosen for IMAT imaging analysis as unique skeletal muscle site for which the accuracy of MRI measurements of muscle tissue composition with corresponding histology in vivo was assessed [12]. The L3–L4 level was selected for the analysis because the muscle cross-sectional area has previously described as the largest overall at this level [26]. IMAT was defined as AT area visible between muscle groups and beneath the muscle fascia (Fig. 1 D).

A high-resolution T1-weighted TSE sequence was obtained and the scanning parameters were TR = 100 ms, TE = 8 ms, slice thickness  = 3 mm, FOV = 256 mm. The gray-level intensity (threshold value) of the AT in the SAT region was determined and used as a reference [27]. This threshold value was reduced by 20% to identify the IMAT threshold (SliceOmatic software).

#### IAAT and SAT areas

IAAT is defined as intraabdominal fat bound by parietal peritoneum or transversalis fascia, excluding the vertebral column and the paraspinal muscles; SAT is fat superficial to the abdominal and back muscles. A breath-hold sequence was used to minimize the effects of respiratory motion on the images. A single image, located at L3–L4 level, obtained using a T1-weighted FFE pulse sequence (TR = 97 ms, TE = 4.6 ms, slice thickness  = 5 mm, FOV = 445 mm), was chosen to assess IAAT area, previously validated as good predictor of total IAAT volume [28] (Fig. 1 E). The same sequence parameters except for FOV = 256 mm were used to measure SAT area at L4–L5 level [29] (Fig. 1 F).

### Statistical analysis

Statistical analysis was performed with the SPSS 19.0 software (SPSS, Chicago). Descriptive statistics were given by means ± SD. The Kolmogorov-Smirnov test was used to verify quantitative variables for normality distribution and non-normal distributed parameters were logarithmically transformed before being used in the subsequent parametric procedures. Comparisons between groups were made using Student's unpaired *t*-test. Relationships between continuous variables were evaluated using Pearson partial correlations including gender, waist circumference and IAAT as potential confounders and resulting β coefficients were provided to evaluate the strengths of the associations. For all these analysis a p-value <0.05 based on two-sided test was considered statistically significant.

## Results

The study population was constituted by 18 men and 14 women, aged 48.5±14 years, with mean BMI of 25.6±3.9 Kg/m^2^ and waist circumference of 103.5±14.8 cm. Whereas no gender-related differences were seen in EAT and IAAT depots, IMAT was significantly more represented in women than in men (W: 925.4±491.3 mm^2^; M: 501.1±337 mm^2^; p<0.05).

All fat depots were positively correlated with both age and waist circumference (Table 1), but the correlation's coefficients revealed distinct patterns. Indeed, IMAT and EAT were more strongly associated with age than waist circumference, whereas SAT followed an opposite trend, and IAAT resulted similarly associated with both. The same distinct patterns were observed when looking at the correlations of fat depots with BMI (IMAT: β = 0.47, p<0.01; EAT: β = 0.46, p<0.01; IAAT: β = 0.63, p<0.0001; SAT: β = 0.77, p<0.0001) instead of waist circumference.

**Table 1:** Gender-adjusted correlations of fat depots with age and waist circumference.

	*Age*		*Waist circumference*	
	β	*p value*	β	*p value*
**IMAT area**	0.63	*0.0001*	0.55	*0.001*
**EAT volume**	0.67	*0.0001*	0.49	*0.004*
**IAAT area**	0.68	*0.0001*	0.66	*0.0001*
**SAT area**	0.40	*0.02*	0.84	*0.0001*

*EAT* epicardial adipose tissue, *IMAT* intermuscular adipose tissue, *IAAT* intraabdominal adipose tissue, *SAT* subcutaneous adipose tissue.

**Figure 2:**
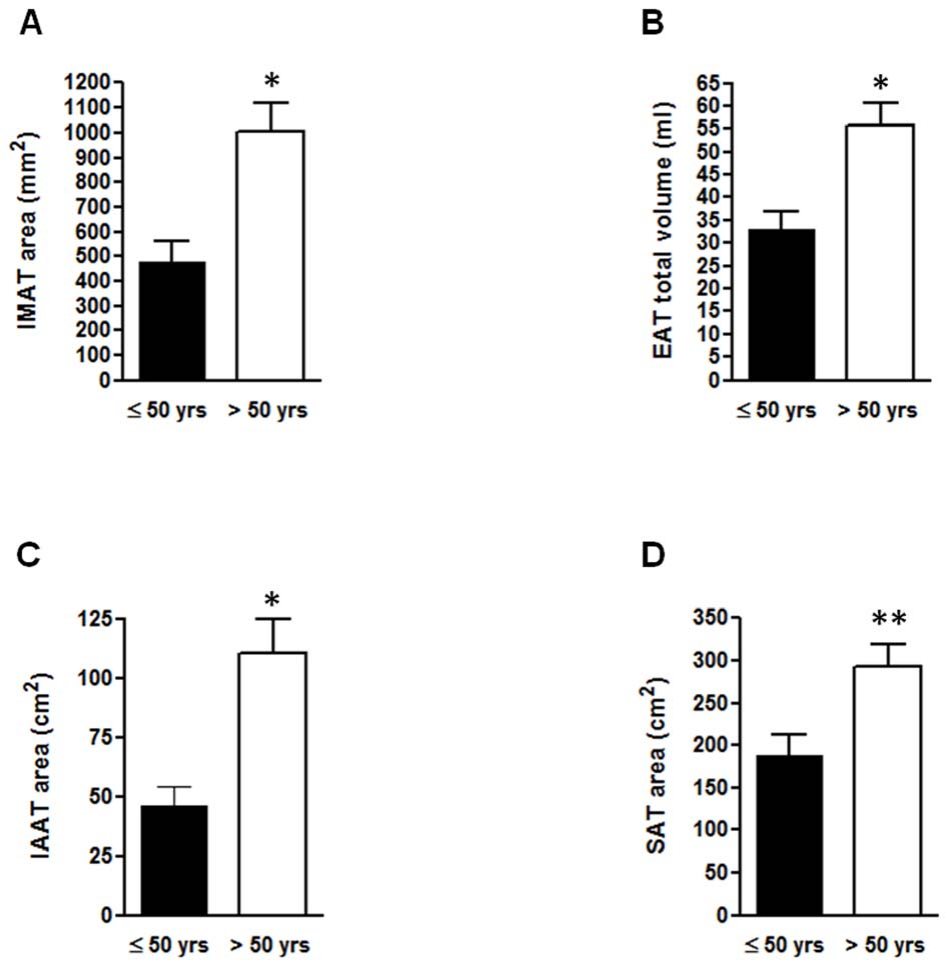
Adipose tissue depots amounts according to age. Erector spinae IMAT area (A), EAT total volume (B), IAAT (C) and SAT (D) areas in subjects ≤50 years and >50 years. *p<0.001, **p<0.05.

We therefore divided our sample according to age (≤50 and >50 years), resulting in two subgroups significantly different for age (p<0.001) and anthropometric measures, so that the older ones had concomitantly higher waist circumference (p<0.01) and BMI (p<0.001) than the youngers (Table 2). Similarly, IMAT, EAT, IAAT and SAT amounts were significantly more represented in older subjects compared to the younger ones (Fig. 2 A–D).

**Table 2:** Study population characteristics according to age.

Clinical Features			
	≤50 yrs	>50 yrs	*p*
N	19	13	
Gender (F/M)	7/12	7/6	
Age (yrs)	38.4±5.2	63.2±8.3	*<0.0001*
Waist circumference (cm)	97.1±13	112.9±12.3	*<0.01*
BMI (Kg/m^2^)	23.8±3.4	28.2±3	*<0.001*
Systolic BP (mmHg)	121.8±11.2	133±11	*<0.01*
Diastolic BP (mmHg)	76±7.5	81.9±7.7	*<0.05*
Glucose (mg/dl)	92.5±9	107.9±31.8	*ns*
Triglycerides (mg/dl)	93.1±46.4	127.7±47.1	*ns*
HDL-Chol (mg/dl)	48.1±12.1	55.9±20.2	*ns*
IFG/T2D	2/0	2/2	
MS	3	8	
Hypolipidemic TH	0	5	

*BMI* body mass index, *BP* blood pressure, *EAT* epicardial adipose tissue, *IFG* impaired fasting glucose, *IMAT* intermuscular adipose tissue, *IAAT* intraabdominal adipose tissue, *MS* metabolic syndrome, *SAT* subcutaneous adipose tissue, *T2D* type 2 diabetes, *TH* therapies.

*significantly different (p<0.05). Data are expressed as mean ± SD.

**Figure 3:**
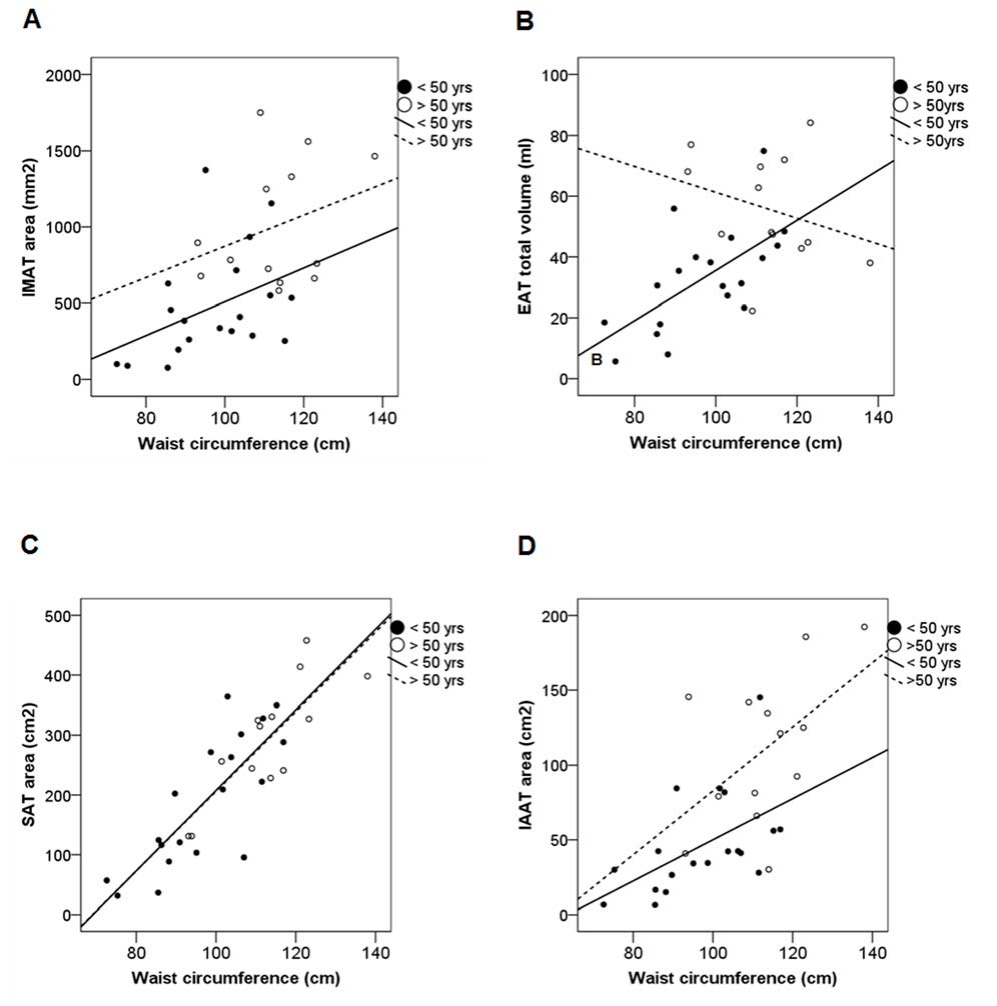
Correlations of adipose tissue depots with waist circumference according to age. Correlations of IMAT area (A), EAT total volume (B), SAT (C) and IAAT (D) areas with waist circumference. Solid lines represent correlations in younger subjects (≤50 years): IMAT: β = 0.46, p = 0.03; EAT: β = 0.63, p = 0.005; SAT: β = 0.81, p = 0.0001; IAAT: β = 0.51, p = 0.02 (gender-adjusted correlation coefficients). Dashed lines represent correlations in older subjects (>50 years): IMAT: β = 0.14, p = ns; EAT: β = −0.13, p = ns; IAAT: β = 0.54, p = 0.09; SAT: β = 0.76, p = 0.002.

Even though the older subjects enrolled in our sample study were characterized by an overall greater amount of fat mass compared to the younger ones, as reflected by differences in MRI assessments and anthropometric parameters, the subsequent regression analysis showed that IMAT and EAT quantitative amounts were positively gender-adjusted associated with waist circumference, but in younger subjects only (p<0.05 for both) (Fig. 3 A,B). On the other hand, SAT and IAAT quantitative depots showed great linear relationship with waist circumference in both younger and older subjects (Fig. 3 C,D), even though the strength of these associations was unexpectedly non significant for IAAT in the older ones (p = 0.09).

In accordance to what observed with waist circumference measures, both IMAT and EAT depots turned out to be gender-adjusted associated with IAAT in youngers (IMAT: β = 0.57, p<0.05; EAT: β = 0.6, p<0.01), but not in subjects >50 years. Interestingly, IMAT and EAT were not associated with SAT.

**Figure 4:**
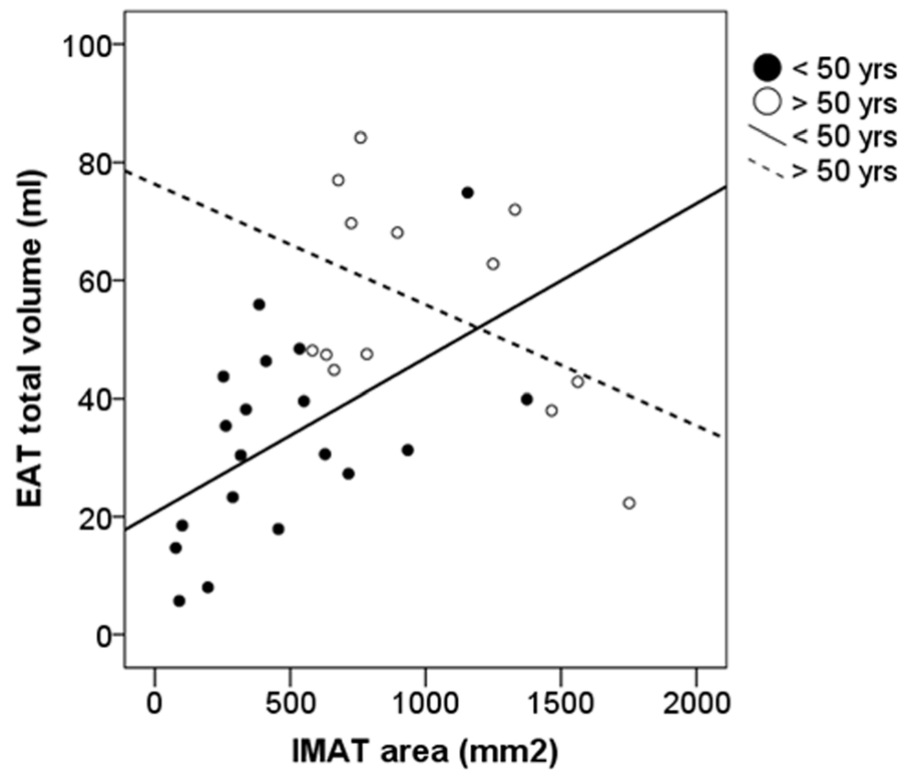
Correlation of IMAT with EAT according to age. Correlations between IMAT area with EAT total volume. Solid lines represent the correlation in younger subjects (≤50 years) (β = 0.6, p = 0.007). Dashed lines represent the non significant correlation in older subjects (>50 years) (β = −0.3, p = ns).

Of interest, the quantitative amounts of IMAT and EAT were significantly correlated with each other, but once again in younger subjects only (β = 0.6, p<0.01) (Fig. 4), whereas the same pattern of relationship was not observed in the olders.

Finally, we observed that IMAT (β = 0.46, p<0.01) and IAAT (β = 0.56, p<0.001), but not EAT and SAT, were positively correlated with serum triglycerides, after adjusting for hypolipidemic therapies. However, after adjusting for either waist circumference or IAAT, the correlation between IMAT and triglycerides was no more significant (waist; β = 0.23, p = 0.2; IAAT: β = 0.19, p = 0.3). Accordingly, patients with MS, who had greater IAAT (MS: 101.3±17.5 cm^2^; no-MS: 57±9.2 cm^2^; p<0.05), SAT (MS: 290.4±31.3 mg/dl; no-MS: 199.1±24.3 mg/dl; p<0.05), fasting glucose (MS: 110.2±9.6 mg/dl; no-MS: 92±2.2 mg/dl; p<0.05) and triglycerides levels (MS: 141.4±12.2 mg/dl; no-MS 88.6±10.2 mg/dl; p<0.01), showed a similar, but not significant, trend for IMAT (MS: 848.3±128 mm^2^; no-MS: 609.8±100 mm^2^; p = ns) and no difference in EAT (MS: 47.1±5.7 ml; no-MS: 39.8±4.6 mm^2^; p = ns) amounts compared to subjects without MS.

## Discussion

In the present study we observed that IMAT, as well as EAT, quantitative amounts are more strongly related with age than waist circumference in sedentary individuals, and that in younger subjects only (≤50 yrs in our study population) a positive association of IMAT depots with waist circumference measures and IAAT amount can be postulated. In addition, no significant and independent relationships were observed between ectopic quantitative depots (IMAT and EAT) and circulating triglycerides levels. Even though in our study population IMAT depots were shown to be more represented in women than in men (p<0.05), the associations reported above turned out to be not influenced by gender. We hypothesize that such a gender-difference in IMAT amount could have been driven by chance, since mid-calf and total body IMAT have been previously reported to be equally distributed among genders [20], [30] and, in addition, no significant gender-related differences in EAT, IAAT and SAT amounts were detected in our sample study.

Even though IMAT could be conceivably more expanded with increasing age, some previous studies have reported conflicting results for this association [16]. Plausible explanations for such a discrepancy might rely on the fact that most studies in the field have failed to account for activity levels and disease conditions or, alternatively, have investigated on subjects in an extremely narrow range of age. Consequently, as ectopic AT depots are known to be strongly influenced by exercise, so that high physical activity is proven to reduce IMAT amount both in younger and older master athletes [18], we decided to apply no age restriction among enrollment criteria of our study population, as well as to focus on well established sedentary subjects in order to avoid this above reported potential confounder on our analysis.

Interestingly, IMAT and EAT depots appeared to be more strongly associated with age than waist circumference or BMI, whereas similar trends were not reported for either IAAT or SAT (Table 1).

The reasons for these findings are not completely understood, but it can be postulated that the distinct origin of ectopic fat, which consists in the dysdifferentiation of muscle mesenchymal progenitors, or the close anatomic contact of AT with muscular fibers, which favors paracrine effects of myokines (IL-6, myostatin, follistatin) and metabolites on the biology of the surrounding AT, could account for distinct behaviors of intermuscular and epicardial adipocytes comparing with those constituting IAAT and SAT [11].

Our observations are in line with the notion that, with increasing age, body fat becomes centralized and is redistributed from subcutaneous to visceral compartments, even in healthy people [31], [32], suggesting that this redistribution could additionally involve ectopic sites such as IMAT and EAT depots and therefore lead to their greater expansion. Accordingly, ageing-associated changes in AT distribution are a well-established phenomenon irrespective of gender or race, accompanied by an increased risk of MS, AT chronic inflammation and decreased proliferation and differentiation of preadipocytes [33]. In addition, as older adults are often weaker than predicted by sarcopenia alone [34] and high levels of leg IMAT are associated with decreased muscle strength as well as muscle quality [16], the increase of fat within skeletal muscle is likely to contribute further to physical impairment and disability in the elderly. Similarly to EAT, where the lack of interposed fascia allows pro-inflammatory [35], cardiodepressant [36] and pro-fibrotic [37] factors to affect myocardial structure and function, IMAT may promote myosteatosis, myofibrosis [38] and functional impairment, by secreting paracrine mediators and enhancing lipolysis rates within skeletal muscle. Accordingly, both IMAT and Tumor Necrosis Factor-α mRNA levels are increased in paretic limbs of stroke survivors [39].

Noteworthy, we also observed that IMAT and EAT amounts positively correlated with waist circumference and reciprocally irrespective of gender, but in younger individuals (≤50 yrs) only, in contrast with what noted for SAT and IAAT which tended to correlate similarly with waist circumference in both age groups (≤50 yrs and >50 yrs).

Even though all recruited subjects did not practice structured or routine exercise, some evidence suggests that even changing the way a muscle is used may play an important role in the level of fatty infiltration seen with ageing: for instance, brisk walking, which is less likely to be practiced by the olders, can decrease IMAT amount [17]. Besides, the higher loss percentage of IAAT compared to IMAT and EAT after exercise-induced weight loss may reflect distinct lipolytic properties [40], [41], and allows to speculate that ectopic compartments also respond differently to inactivity.

We could additionally postulate that these age-related different patterns could be consequent to modifications in sex hormones levels, chronic stress-induced mild hypercortisolemia and prolonged sympathetic nervous system activation, or even to nutritional factors, across the ages [3]. With regard to the latter, fructose-sweetened beverages consumption may have a potential effect on body fat distribution independent of its impact on overall AT accretion, namely stimulating deposition of triglycerides in ectopic sites by depot-specific modulation of lipogenic enzymes [42].

Due to its association with age [15], physical inactivity [18], adiposity [43], muscle function impairment [16] and type 2 diabetes, IMAT has been suggested to be a risk factor for obesity-related diseases along with IAAT. The association with insulin resistance and glucose metabolism has been previously reported for erector spinae, thigh and total body IMAT [20], [38], [40], [44], [45] irrespective of race, weight, height, and total skeletal muscle volume [19]. Furthermore, thigh IMAT was significantly associated with increased systemic levels of inflammatory cytokines and C-Reactive Protein, which have been linked to insulin resistance, type 2 diabetes and age-related sarcopenia [46], [47].

Nevertheless, it is currently unknown whether IMAT can play a modifying role in the pathogenesis of insulin resistance or it is to be alternatively considered a marker of metabolic dysfunction only. The finding that IMAT amounts were positively correlated with serum triglycerides, even though no causal relationship can be affirmed, could suggest a contribution of IMAT in determining obesity- and age-related metabolic diseases.

In accordance, IMAT is assumed to modulate blood flow to the muscle, impair insulin diffusion capacity [20], [48], and increase local inflammation and lipolysis rates within the muscle. However, after controlling for waist circumference or IAAT, the correlation between IMAT and triglycerides was no more significant, highlighting the greater relevance of IAAT in directly influencing the metabolism, putatively through its greater extent and unique anatomic location, which allow the release of free fatty acids into the portal system to the liver and pancreas.

Finally, we acknowledge some study limitations, mainly related to the cross-sectional design which does not allow to assess cause-effect relationships, and to the small sample size which precludes to address other potential confounders using covariate analysis, e.g. the interaction between age and waist circumference in their relationships with IMAT or EAT. The study is also adversely affected by the lack of any direct measure of insulin resistance, which would have been useful to better characterize our study population from a metabolic perspective, although the presence of MS diagnosed according to IDF criteria can provide indirect information about insulin resistance status [49]. On the other hand, we believe that major strengths of our study derive from the use of MRI, namely the gold standard technique to assess AT quantitative distribution and from the opportunity to include individuals with known sedentary habits and in a wide distribution of age.

In summary, herein we report that in sedentary subjects without cardiovascular disease IMAT as well as EAT depots are related with increasing age to a greater extent than observed with waist circumference, and that the positive association of IMAT amount with waist circumference and IAAT is likely to be significant for younger individuals only. Further research is needed to better clarify these issues and, more generally, to better characterize the role of ectopic AT depots in the pathophysiology of cardiometabolic diseases.

## References

[1] Fox CS, Massaro JM, Hoffmann U, Pou KM, Maurovich-Horvat P, et al (2007) Abdominal visceral and subcutaneous adipose tissue compartments: association with metabolic risk factors in the Framingham Heart Study. Circulation 116: 39–48. 17576866 10.1161/CIRCULATIONAHA.106.675355

[2] Jensen MD (2008) Role of body fat distribution and the metabolic complications of obesity. J Clin Endocrinol Metab 93: S57–63. 18987271 10.1210/jc.2008-1585 PMC2585758

[3] Tchernof A, Despres JP (2013) Pathophysiology of human visceral obesity: an update. Physiol Rev 93: 359–404. 23303913 10.1152/physrev.00033.2011

[4] Tchkonia T, Thomou T, Zhu Y, Karagiannides I, Pothoulakis C, et al (2013) Mechanisms and metabolic implications of regional differences among fat depots. Cell Metab 17: 644–656. 23583168 10.1016/j.cmet.2013.03.008 PMC3942783

[5] Thamer C, Machann J, Haap M, Stefan N, Heller E, et al (2004) Intrahepatic lipids are predicted by visceral adipose tissue mass in healthy subjects. Diabetes Care 27: 2726–2729. 15505012 10.2337/diacare.27.11.2726

[6] Thamer C, Machann J, Bachmann O, Haap M, Dahl D, et al (2003) Intramyocellular lipids: anthropometric determinants and relationships with maximal aerobic capacity and insulin sensitivity. J Clin Endocrinol Metab 88: 1785–1791. 12679474 10.1210/jc.2002-021674

[7] Gastaldelli A, Basta G (2010) Ectopic fat and cardiovascular disease: what is the link? Nutr Metab Cardiovasc Dis 20: 481–490. 20659791 10.1016/j.numecd.2010.05.005

[8] Fluchter S, Haghi D, Dinter D, Heberlein W, Kuhl HP, et al (2007) Volumetric assessment of epicardial adipose tissue with cardiovascular magnetic resonance imaging. Obesity (Silver Spring) 15: 870–878. 17426322 10.1038/oby.2007.591

[9] Wang TD, Lee WJ, Shih FY, Huang CH, Chang YC, et al (2009) Relations of epicardial adipose tissue measured by multidetector computed tomography to components of the metabolic syndrome are region-specific and independent of anthropometric indexes and intraabdominal visceral fat. J Clin Endocrinol Metab 94: 662–669. 19050055 10.1210/jc.2008-0834

[10] Silaghi A, Piercecchi-Marti MD, Grino M, Leonetti G, Alessi MC, et al (2008) Epicardial adipose tissue extent: relationship with age, body fat distribution, and coronaropathy. Obesity (Silver Spring) 16: 2424–2430. 18719675 10.1038/oby.2008.379

[11] Vettor R, Milan G, Franzin C, Sanna M, De Coppi P, et al (2009) The origin of intermuscular adipose tissue and its pathophysiological implications. Am J Physiol Endocrinol Metab 297: E987–998. 19738037 10.1152/ajpendo.00229.2009

[12] Rossi A, Zoico E, Goodpaster BH, Sepe A, Di Francesco V, et al (2010) Quantification of intermuscular adipose tissue in the erector spinae muscle by MRI: agreement with histological evaluation. Obesity (Silver Spring) 18: 2379–2384. 20300085 10.1038/oby.2010.48 PMC5278643

[13] Karampinos DC, Baum T, Nardo L, Alizai H, Yu H, et al (2012) Characterization of the regional distribution of skeletal muscle adipose tissue in type 2 diabetes using chemical shift-based water/fat separation. J Magn Reson Imaging 35: 899–907. 22127958 10.1002/jmri.23512 PMC3292710

[14] Gallagher D, Kuznia P, Heshka S, Albu J, Heymsfield SB, et al (2005) Adipose tissue in muscle: a novel depot similar in size to visceral adipose tissue. Am J Clin Nutr 81: 903–910. 15817870 10.1093/ajcn/81.4.903 PMC1482784

[15] Marcus RL, Addison O, Kidde JP, Dibble LE, Lastayo PC (2010) Skeletal muscle fat infiltration: impact of age, inactivity, and exercise. J Nutr Health Aging 14: 362–366. 20424803 10.1007/s12603-010-0081-2 PMC3758242

[16] Goodpaster BH, Carlson CL, Visser M, Kelley DE, Scherzinger A, et al (2001) Attenuation of skeletal muscle and strength in the elderly: The Health ABC Study. J Appl Physiol (1985) 90: 2157–2165. 11356778 10.1152/jappl.2001.90.6.2157

[17] Goodpaster BH, Chomentowski P, Ward BK, Rossi A, Glynn NW, et al (2008) Effects of physical activity on strength and skeletal muscle fat infiltration in older adults: a randomized controlled trial. J Appl Physiol (1985) 105: 1498–1503. 18818386 10.1152/japplphysiol.90425.2008 PMC2584841

[18] Manini TM, Clark BC, Nalls MA, Goodpaster BH, Ploutz-Snyder LL, et al (2007) Reduced physical activity increases intermuscular adipose tissue in healthy young adults. Am J Clin Nutr 85: 377–384. 17284732 10.1093/ajcn/85.2.377

[19] Albu JB, Kovera AJ, Allen L, Wainwright M, Berk E, et al (2005) Independent association of insulin resistance with larger amounts of intermuscular adipose tissue and a greater acute insulin response to glucose in African American than in white nondiabetic women. Am J Clin Nutr 82: 1210–1217. 16332653 10.1093/ajcn/82.6.1210 PMC2670467

[20] Goodpaster BH, Thaete FL, Kelley DE (2000) Thigh adipose tissue distribution is associated with insulin resistance in obesity and in type 2 diabetes mellitus. Am J Clin Nutr 71: 885–892. 10731493 10.1093/ajcn/71.4.885

[21] Gallagher D, Kelley DE, Yim JE, Spence N, Albu J, et al (2009) Adipose tissue distribution is different in type 2 diabetes. Am J Clin Nutr 89: 807–814. 19158213 10.3945/ajcn.2008.26955 PMC2714397

[22] Ross R, Leger L, Morris D, de Guise J, Guardo R (1992) Quantification of adipose tissue by MRI: relationship with anthropometric variables. J Appl Physiol (1985) 72: 787–795. 1559959 10.1152/jappl.1992.72.2.787

[23] Sobol W, Rossner S, Hinson B, Hiltbrandt E, Karstaedt N, et al (1991) Evaluation of a new magnetic resonance imaging method for quantitating adipose tissue areas. Int J Obes 15: 589–599. 1960010

[24] Alberti KG, Zimmet P, Shaw J (2005) The metabolic syndrome–a new worldwide definition. Lancet 366: 1059–1062. 16182882 10.1016/S0140-6736(05)67402-8

[25] Halberg N, Khan T, Trujillo ME, Wernstedt-Asterholm I, Attie AD, et al (2009) Hypoxia-inducible factor 1alpha induces fibrosis and insulin resistance in white adipose tissue. Mol Cell Biol 29: 4467–4483. 19546236 10.1128/MCB.00192-09 PMC2725728

[26] Marras WS, Jorgensen MJ, Granata KP, Wiand B (2001) Female and male trunk geometry: size and prediction of the spine loading trunk muscles derived from MRI. Clin Biomech (Bristol, Avon) 16: 38–46. 10.1016/s0268-0033(00)00046-2 11114442

[27] Song MY, Ruts E, Kim J, Janumala I, Heymsfield S, et al (2004) Sarcopenia and increased adipose tissue infiltration of muscle in elderly African American women. Am J Clin Nutr 79: 874–880. 15113728 10.1093/ajcn/79.5.874

[28] Demerath EW, Shen W, Lee M, Choh AC, Czerwinski SA, et al (2007) Approximation of total visceral adipose tissue with a single magnetic resonance image. Am J Clin Nutr 85: 362–368. 17284730 10.1093/ajcn/85.2.362 PMC2883309

[29] Wajchenberg BL (2000) Subcutaneous and visceral adipose tissue: their relation to the metabolic syndrome. Endocr Rev 21: 697–738. 11133069 10.1210/edrv.21.6.0415

[30] Ruan XY, Gallagher D, Harris T, Albu J, Heymsfield S, et al (2007) Estimating whole body intermuscular adipose tissue from single cross-sectional magnetic resonance images. J Appl Physiol (1985) 102: 748–754. 17053107 10.1152/japplphysiol.00304.2006 PMC2758818

[31] Bazzocchi A, Diano D, Ponti F, Andreone A, Sassi C, et al (2013) Health and ageing: a cross-sectional study of body composition. Clin Nutr 32: 569–578. 23111003 10.1016/j.clnu.2012.10.004

[32] Bazzocchi A, Ponti F, Diano D, Moio A, Albisinni U, et al.. (2014) Abdominal adiposity by ultrasonography: A “pocket” database for reference standard in Italian people. Prim Care Diabetes. 10.1016/j.pcd.2014.02.003 24636921

[33] Sepe A, Tchkonia T, Thomou T, Zamboni M, Kirkland JL (2011) Aging and regional differences in fat cell progenitors - a mini-review. Gerontology 57: 66–75. 20110661 10.1159/000279755 PMC3031153

[34] Kallman DA, Plato CC, Tobin JD (1990) The role of muscle loss in the age-related decline of grip strength: cross-sectional and longitudinal perspectives. J Gerontol 45: M82–88. 2335723 10.1093/geronj/45.3.m82

[35] Sacks HS, Fain JN, Cheema P, Bahouth SW, Garrett E, et al (2011) Inflammatory genes in epicardial fat contiguous with coronary atherosclerosis in the metabolic syndrome and type 2 diabetes: changes associated with pioglitazone. Diabetes Care 34: 730–733. 21289232 10.2337/dc10-2083 PMC3041217

[36] Greulich S, Maxhera B, Vandenplas G, de Wiza DH, Smiris K, et al (2012) Secretory products from epicardial adipose tissue of patients with type 2 diabetes mellitus induce cardiomyocyte dysfunction. Circulation 126: 2324–2334. 23065384 10.1161/CIRCULATIONAHA.111.039586

[37] Venteclef N, Guglielmi V, Balse E, Gaborit B, Cotillard A, et al.. (2013) Human epicardial adipose tissue induces fibrosis of the atrial myocardium through the secretion of adipo-fibrokines. Eur Heart J. 10.1093/eurheartj/eht099 23525094

[38] Zoico E, Corzato F, Bambace C, Rossi AP, Micciolo R, et al (2013) Myosteatosis and myofibrosis: Relationship with aging, inflammation and insulin resistance. Arch Gerontol Geriatr 57: 411–416. 23809667 10.1016/j.archger.2013.06.001 PMC5278642

[39] Hafer-Macko CE, Yu S, Ryan AS, Ivey FM, Macko RF (2005) Elevated tumor necrosis factor-alpha in skeletal muscle after stroke. Stroke 36: 2021–2023. 16109906 10.1161/01.STR.0000177878.33559.fe

[40] Janssen I, Fortier A, Hudson R, Ross R (2002) Effects of an energy-restrictive diet with or without exercise on abdominal fat, intermuscular fat, and metabolic risk factors in obese women. Diabetes Care 25: 431–438. 11874926 10.2337/diacare.25.3.431

[41] Kim MK, Tomita T, Kim MJ, Sasai H, Maeda S, et al (2009) Aerobic exercise training reduces epicardial fat in obese men. J Appl Physiol (1985) 106: 5–11. 18927266 10.1152/japplphysiol.90756.2008

[42] Stanhope KL, Havel PJ (2008) Endocrine and metabolic effects of consuming beverages sweetened with fructose, glucose, sucrose, or high-fructose corn syrup. Am J Clin Nutr 88: 1733S–1737S. 19064538 10.3945/ajcn.2008.25825D PMC3037017

[43] Goodpaster BH, Krishnaswami S, Harris TB, Katsiaras A, Kritchevsky SB, et al (2005) Obesity, regional body fat distribution, and the metabolic syndrome in older men and women. Arch Intern Med 165: 777–783. 15824297 10.1001/archinte.165.7.777

[44] Goodpaster BH, Krishnaswami S, Resnick H, Kelley DE, Haggerty C, et al (2003) Association between regional adipose tissue distribution and both type 2 diabetes and impaired glucose tolerance in elderly men and women. Diabetes Care 26: 372–379. 12547865 10.2337/diacare.26.2.372

[45] Yim JE, Heshka S, Albu J, Heymsfield S, Kuznia P, et al (2007) Intermuscular adipose tissue rivals visceral adipose tissue in independent associations with cardiovascular risk. Int J Obes (Lond) 31: 1400–1405. 17452994 10.1038/sj.ijo.0803621 PMC2752367

[46] Beasley LE, Koster A, Newman AB, Javaid MK, Ferrucci L, et al (2009) Inflammation and race and gender differences in computerized tomography-measured adipose depots. Obesity (Silver Spring) 17: 1062–1069. 19165157 10.1038/oby.2008.627 PMC3268118

[47] Jensen GL (2008) Inflammation: roles in aging and sarcopenia. JPEN J Parenter Enteral Nutr 32: 656–659. 18974248 10.1177/0148607108324585

[48] Boettcher M, Machann J, Stefan N, Thamer C, Haring HU, et al (2009) Intermuscular adipose tissue (IMAT): association with other adipose tissue compartments and insulin sensitivity. J Magn Reson Imaging 29: 1340–1345. 19422021 10.1002/jmri.21754

[49] Anderson PJ, Critchley JA, Chan JC, Cockram CS, Lee ZS, et al (2001) Factor analysis of the metabolic syndrome: obesity vs insulin resistance as the central abnormality. Int J Obes Relat Metab Disord 25: 1782–1788. 11781758 10.1038/sj.ijo.0801837

